# Antioxidant and Antibacterial Peptides from Soybean Milk through Enzymatic- and Membrane-Based Technologies

**DOI:** 10.3390/bioengineering7010005

**Published:** 2019-12-28

**Authors:** Arijit Nath, Geremew Geidare Kailo, Zsuzsanna Mednyánszky, Gabriella Kiskó, Barbara Csehi, Klára Pásztorné-Huszár, Renáta Gerencsér-Berta, Ildikó Galambos, Emília Pozsgai, Szilvia Bánvölgyi, Gyula Vatai

**Affiliations:** 1 Department of Food Engineering, Faculty of Food Science, Szent István University, Ménesi st 44, HU-1118 Budapest, Hungary; arijit0410@gmail.com (A.N.);; 2 Soós Ernő Water Technology Research and Development Center, University of Pannonia, Zrínyi M. u. 18, H-8800 Nagykanizsa, Hungary; 3 Department of Food Chemistry and Nutrition, Faculty of Food Science, Szent István University, Budapest, Somlói st 14-16, HU-1118 Budapest, Hungary; 4 Department of Food Microbiology and Biotechnology, Faculty of Food Science, Szent István University, Budapest, Somlói st 14-16, HU-1118 Budapest, Hungary; 5 Department of Refrigeration and Livestock Product Technology, Faculty of Food Science, Szent István University, Ménesi st 43-45, HU-1118 Budapest, Hungary; Csehi.Barbara@etk.szie.hu (B.C.); Pasztorne.Huszar.Klara@etk.szie.hu (K.P.-H.)

**Keywords:** soybean milk, antioxidant peptide, antibacterial peptide, enzymatic hydrolysis, membrane bioreactor

## Abstract

Enzymatic hydrolysis of soybean milk proteins with cysteine protease papain was performed in an advanced bioreactor, operated with batch mode. In soybean milk protein hydrolysis reaction, enzyme and substrate ratio and reaction temperature were varied, ranging from 0.029:100–0.457:100 and 30–60 °C, respectively. The degree of hydrolysis of soybean milk proteins was increased with increase of enzyme and substrate (soybean milk protein) ratio. However, the degree of hydrolysis was increased due to change of reaction temperature from 30 °C to 60 °C with enzyme and substrate ratio 0.229:100 and was reduced when hydrolysis reaction was performed with enzyme and substrate ratio 0.11:100 at hydrolysis temperature 60 °C. Antioxidant capacity of enzyme-treated milk had a similar trend with degree of hydrolysis. In a later exercise, a membrane bioreactor was adopted for continuous production of antioxidant and antibacterial peptides from soybean milk. The membrane bioreactor was operated for 12 h with constant feeding. Ceramic-made tubular membrane with a pore size 20 nm was used. Application of static turbulence promoter in a membrane separation process was investigated and its positive effects, with respect to higher permeate flux and lower energy consumption in filtration process, were proven. Antioxidant capacity and antibacterial activity against *Bacillus cereus* of enzyme-hydrolyzed milk and permeate from membrane were confirmed.

## 1. Introduction

Soybean (*Glycine max*) has long been recognized as a source of high-quality protein and is considered the largest edible protein source around the world [1]. Prior to this, soybean was considered as a feed stock of edible oil, lactose-free milk formula and in a variety of main and side dishes, such as tempeh, natto, sauce, miso, douchi, kinema, cheonggukjang, doenjang, kanjang, gochujang, yoghurt, and okara [2]. Beside the preparation of soybean-based foods, synthesis, isolation, and characterization of peptides from soybean protein have grabbed lots of attention due to their unique biochemical and medicinal importance [3,4]. Peptides from soybean may produce through acid hydrolysis, microwave-assisted process, and ultra-heat treatment. Preparation of peptides from soybean through a biological route (microbial fermentation and enzymatic hydrolysis) is referred to as “generally regarded as safe” [5]. Furthermore, it has been proven that enzymatic hydrolysis of proteins may often generate new antigenic substances, which play a role in immunomodulation [6]. Several researches address development of bioactive peptides from soybean meal [7,8,9], soybean protein concentrate [10,11], soybean protein isolate [11,12,13,14,15,16], soy flour [17,18,19], and soybean protein hydrolysate [20,21] through serine protease, which generally produces peptides with a bitter taste [22,23,24]. Application of cysteine protease in the food industry is noteworthy because it does not create undesirable organoleptic property of food products [25,26,27]. Unfortunately, studies about bioactive peptides from soybean milk with papain, a member of cysteine protease, is scanty. In most cases, biotransformation of soybean proteins through enzymatic route in a bioreactor, operated with batch mode [7,9,10,12,13,14,17,18,19], and subsequent purification with single or a cascade of membrane filtration [8,9,15,16,21] and chromatographic processes [14,21] have been considered. Considerable drawbacks for the mentioned technologies might be (a) high space time due to sequential enzymatic reaction and purification steps, (b) overall high process footprint with lower throughput, (c) high cost of enzyme and less chance to reuse enzyme due to its inactivation, and (d) isolation of specific bioactive peptide from neighborhood molecules. Without any contradiction, it may be that the membrane bioreactor may be an acceptable choice to solve the mentioned drawbacks. Based on a literature review, angiotensin I-converting inhibitory peptides are produced from isolated soybean protein [28,29] and antimicrobial peptides from defatted soybean meal [8] by membrane bioreactor. Challenges related to the membrane bioreactor are (a) lower permeate flux due to formation of concentration polarization, (b) membrane fouling, and (c) high operating cost [30,31]. There is therefore an urgent need to modify the conventional membrane bioreactor or explore a tool that can reduce the limitation of membrane bioreactor and boost its application for production of foods and biopharmaceuticals.

Realizing the unique biological activity of soybean-derived peptides, the primary objective of investigation was to understand the feasibility of preparing antioxidant and antibacterial peptides from soybean milk through enzymatic and membrane processes. The second objective of investigation was intensification of membrane bioreactor for continuous production of antioxidant and antibacterial peptides from soybean milk. Initially, enzymatic hydrolysis of soybean milk proteins was performed in an advanced-controlled bioreactor, operated with batch mode. In a later exercise, antioxidant and antibacterial peptides from soybean milk were produced by membrane bioreactor, operated with a continuous mode. In a previous investigation, we found that peptides with molecular weight lower than 5 kDa, produced from defatted soybean meal with trypsin, offered antibacterial activity against *Bacillus cereus.* Therefore, in the present investigation, a tubular ceramic membrane with pore size of 20 nm, which allows permeation of peptides, and with molecular weight lower than 5 kDa was used in membrane separation unit [8]. Application of static turbulence promoter in the membrane separation process was investigated and it was proven that static turbulence promoter may intensify the performance of membrane bioreactor. It offered higher permeate flux with lower energy consumption in the filtration process. Antioxidant capacity and antibacterial activity against *Bacillus cereus* of enzyme-hydrolyzed milk and membrane permeate were confirmed. 

## 2. Materials and Methods 

### 2.1. Chemicals and Reagents

Lyophilized papain (~30,000 USP units/mg) was purchased from Himedia, India. Sodium acetate (anhydrous, ≥99%), trichloroacetic acid (≥99%), and 2,4,6-Tris(2-pyridyl)-s-triazine (≥98%) were obtained from the Sigma-Aldrich group (Schnelldorf, Germany). Citric acid (99%), potassium sulfate (≥99%), copper sulfate (≥99%) sulfuric acid (≥99%), phenolphthalein (≥98%), methanol (≥98%), glucose (≥99%), phenol (≥99%), and sodium hydroxide (≥99%) were purchased from Reanal (Budapest, Hungary). Ultrasil P3-11 was purchased from Ecolab-Hygiene Kft (Budapest, Hungary). Ferric chloride (≥99%), sulfuric acid (≥99.9%), amyl alcohol (≥99.9%), ascorbic acid (99.7%), bacteriological agar powder, and soybean casein digestive medium were procured from Merck (Darmstadt, Germany). Acrylamide (≥99%), sodium-dodecyl sulfate (≥99%), ammonium persulfate (≥99%), Tetramethylethylenediamine (≥99%), tris(hydroxymethyl)aminomethane (≥99%), glycine (≥99%), ethyl alcohol (≥99%), coomassie blue stain (≥99%), acetic acid (≥99%), isopropanol (≥99%), glycerol (≥99%), 2-βmercaptoethanol (≥99%), and bromophenol blue (≥99%) were purchased from BIO-RAD (BIO-RAD, USA). Milli-Q ultrapure deionized water (18.2 MΩ·cm; Merck-Millipore, Molsheim, France) was used throughout the experiment.

### 2.2. Membrane Bioreactor 

**Figure 1:**
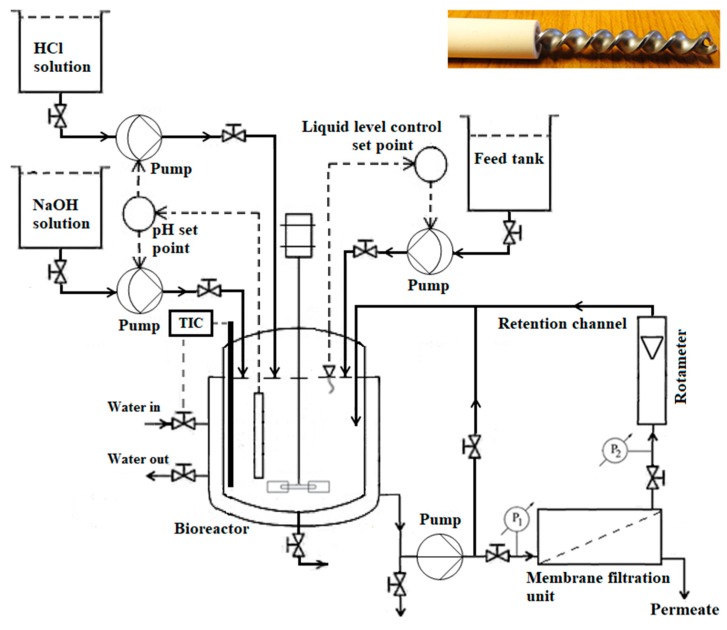
Schematic diagram of membrane bioreactor (inset: Ceramic tubular membrane with twisted tape static turbulence promoter).

An in-house developed membrane bioreactor, i.e., a bioreactor with an external membrane separation unit, was adopted for preparation of antioxidant and antibacterial peptides with molecular weight lower than 5 kDa from soybean milk. An advanced-controlled bioreactor with an aspect ratio (height:diameter) of 2:1 and working volume 0.8 L (Solida Biotech, München, Germany) was fitted with a cross-flow single-channel tubular membrane module, made of stainless steel (SS316). Temperature measuring sensor and pH meter were placed inside the bioreactor to measure temperature and pH, respectively, during enzymatic reaction. The bioreactor had a water jacket and it was fitted with a thermostat to maintain constant temperature during enzymatic reaction. Trans-membrane pressure of membrane module was controlled by pressure gauges and flow control valves, fitted at two opposite ends of membrane module. Feed flow rate for membrane module was controlled using a centrifugal pump (Hydra-Cell G03; Verder Hungary Kft, Budapest, Hungary). A rotameter at retentate end and a bypass valve were also used to control the flow rate in membrane module. The schematic diagram of membrane bioreactor is represented in Figure 1.

Inside of the membrane module, a tubular ceramic ultrafiltration membrane (Membralox^®^ T1-70; Pall Corporation, Dreieich, Germany) was placed. A stainless-steel-made twisted tape static turbulence promoter was placed inside the membrane tube. Detailed specification of membrane and static turbulence promoter are represented in Table 1.

### 2.3. Preparation of Soybean Milk 

Soybean milk was prepared before each experiment (n = 40). Initially, 250 g of water-cleaned soybean seeds were soaked in 1 L of deionized water for 24 h at room temperature (~25 °C). Subsequently, soaking water was drained. In each batch, 500 g of beans were grounded with 800 mL of deionized water, pH 7, with a laboratory blender (VWR 58985-012; VWR International, Radnor PA, USA) for 10 min. After blending of soaked-soybean seeds, viscous solution was created. It was filtered through muslin cloth by pressing and solid was separated. Average value of the concentration of total protein and pH in filtrate from muslin cloth, obtained from different batches were 40 ± 0.96 g/L and 7.1 ± 0.15, respectively. Subsequently, pH of filtrate from muslin cloth was adjusted to 7.0 by 2.0 N of hydrochloric acid or sodium hydroxide [32]. After adjustment of pH, concentration of total protein in soybean milk was adjusted to 28 ± 0.18 g/L with sterile deionized water, pH 7. With the mentioned protocol, 700 mL of soybean milk was finally achieved from 500 g of soaked soybean seeds. Developed soybean milk was stored in sterilized screw cap bottle at temperature 4 °C in a refrigerator for subsequent experimental purpose. It was considered as native (unhydrolyzed) soybean milk.

### 2.4. Enzymatic Hydrolysis of Soybean Milk Protein in Bioreactor

Functional properties of peptides, such as antioxidant capacity and antimicrobial activity, may be predicted by their molecular weight, net charge, amino acid sequence, hydrophilicity, and hydrophobicity. Bioavailability and functional properties of peptides from proteins depend on type of enzyme used for proteolysis, degree of hydrolysis, and processing conditions [24]. Enzymatic hydrolysis of soybean milk proteins was performed using papain in a laboratory-scale well-equipped bioreactor (Solida Biotech, München, Germany). Individual batch-mode enzymatic hydrolysis experiment was performed with 500 mL of soybean milk, pH 7. To prepare enzyme solution, 0.09 g of lyophilized papain (non-immobilized) was dissolved in 10 mL of sterile de-ionized water, pH 7, in aseptic condition and filtered through 0.22 μm of PTFE syringe filter (VWR International, Radnor PA, USA). There was no loss of papain (according to concentration of total protein) in filtrate from PTFE syringe filter. Concentration of total protein in solution from syringe filter was determined by the Kjeldahl method [33]. Prior to enzymatic reaction, concentration of protein in soybean milk was also measured by the Kjeldahl method [33] and soybean milk was pre-incubated to reach the desired reaction temperature. After pre-incubation of soybean milk, 450 µL, 900 µL, 1.8 mL, 3.6 mL, and 7.2 mL of enzyme solution from stock solution were aseptically injected to 500 mL of soybean milk in bioreactor. As a result, enzyme and substrate (soybean milk proteins) ratio was varied, such as 0.02:100 (S.D. ± 1.85 × 10^−4^), 0.057:100 (S.D. ± 3.69 × 10^−4^), 0.114:100 (S.D. ± 7.39 × 10^−4^), 0.229:100 (S.D. ± 1.58 × 10^−3^), and 0.457:100 (S.D. ± 2.95 × 10^−3^). Therefore, concentration of papain in soybean milk was ~0.008, ~0.016, ~0.032, ~0.064, and ~0.128 g/L. Enzyme and substrate ratio was calculated according to amount of enzyme (in gram) in to 100 g of substrate (soybean milk protein). Furthermore, effect of reaction temperature, ranging from 30–60 °C, was studied in enzymatic hydrolysis reaction. Soybean milk was treated with enzyme for 10 min at operational temperature and constant agitation with 175 rpm. During enzymatic hydrolysis reaction, pH of soybean milk in bioreactor was maintained constant 7.0 by automated addition of 2.0 N of sodium hydroxide or hydrochloric acid [32]. After 10 min of enzymatic hydrolysis reaction, 15 mL of sample was withdrawn from the bioreactor through a syringe and was collected in a sample tube. Sample tubes were immediately placed in a water bath (Precision COL 19; Thermo ScientificTM, Waltham MA, USA) at temperature 70 °C for 30 min to inactivate the activity of enzyme. 

### 2.5. Estimation of Degree of Hydrolysis of Soybean Milk Protein

Degree of hydrolysis was estimated by amount of TCA-soluble nitrogen in enzymatic reaction mixture after hydrolysis reaction and inactivation of enzyme [34]. After inactivation of enzyme, temperature of sample was reduced to room temperature (~25 °C) and 5 mL of sample was treated with 5 mL of 10% of trichlorocacetic acid (TCA) at a temperature of ~25 °C for 30 min [35,36]. Subsequently, the sample was centrifuged with 5000 rpm (Z206A; Hermle, Gosheim, Germany) at a temperature of 4 °C for 10 min. Supernatant was clarified by Whatman filter paper with pore size 0.2 µm. Concentration of protein in the supernatant was determined by the Kjeldahl method [33]. Degree of hydrolysis (DH) of soybean milk protein was calculated according to the following equation:

(1) $$\%\mathrm{DH}=\frac{TCA\;soluble\;N_{2}\;after\;hydrolysis-TCA\;soluble\;N_{2}\;before\;hydrolysis}{Total\;N_{2}\;in\;soybean\;milk-TCA\;soluble\;N_{2}\;before\;hydrolysis}\times 100[-].$$

### 2.6. Production of Antioxidant and Antibacterial Peptides by Membrane Bioreactor

Antioxidant and antibacterial peptides were produced from soybean milk by an in-house-developed membrane bioreactor, operated with continuous mode. In this case, it was considered that antioxidant and antibacterial peptides were passed through the porous channel of ultrafiltration membrane as permeate, whereas papain (molecular weight ~23 kDa) and residual high-molecular weight peptides (molecular weight higher than 5 kDa) were rejected by the membrane. Prior to starting the membrane bioreactor with continuous-mode operation, membrane compaction was performed with trans-membrane pressure 4 bar and retentate flow rate 200 L/h, until the water flux became steady. Subsequently, the remaining water within dead volume of membrane module was removed carefully. Soybean milk was preincubated at a temperature of 50 °C in the bioreactor. Similar to before, 0.9 g lyophilized papain (non-immobilized) was dissolved in 100 mL of sterile de-ionized water in aseptic condition and filtered through 0.22 μm of PTFE syringe filter (VWR International, Radnor PA, USA). The process was started with 720 mL of soybean milk (initial volume), pH 7, and 80 mL of sterile enzyme solution. Therefore, it may be said that the membrane bioreactor was started with 0.9 g/L of papain and 25 g/L of soybean milk protein. In the bioreactor, constant stirring with 150 rpm and a temperature of 50 °C were maintained throughout the experiment [37,38]. Membrane permeates with constant volume were collected in fraction basis and the volume level of soybean milk in the bioreactor was maintained constant through level-controller and constant supply of enzyme-free soybean milk. During the whole process, pH of soybean milk in bioreactor was maintained at 7.0 by automated addition of 2.0 N of hydrochloric acid or sodium hydroxide [32]. In the membrane separation unit, constant operational trans-membrane pressure of 3 bar and retentate flow rate of 100 L/h were maintained carefully. The effect of static turbulence promoter in filtration process was investigated. Permeate flux (*J*) was calculated based on following equation.

(2) $$J=\frac{V}{A\times t}\;\left[\frac{L}{m^{2}\times h}\right],$$

where *V* = Volume of permeate (L), *A* = Active membrane surface area (m^2^), *t* = Filtration time (h), and *J* = Permeate flux (L/(m^2^ h)) [39].

### 2.7. Specific Energy Consumption of Membrane Filtration

Pressures at two opposite ends of the membrane module were recorded, and the pressure drop (Δ*p*) was calculated from that information. Specific energy consumption (*E*_s_) during filtration process was calculated by the following equation.

(3) $$E_{s}=\frac{Q_{R}\times \Delta p}{J_{s}\times A}\;\left[\frac{\mathrm{kW}\;h}{m^{3}}\right],$$

where *E*_s_ = Specific energy consumption (kW h/m^3^), *Q*_R_ = Retentate flow rate of membrane module (L/h), Δ*p* = Pressure drop (Newton/m^2^), *A* = Active membrane surface area (m^2^), and *J_s_* = Steady state permeate flux (L/(m^2^ h)). During the conversion of unit, 1 Nm = 2.778 × 10^−7^ kW h was considered [40]. 

### 2.8. Membrane Cleaning 

After the experiment, ultrafiltration membrane was cleaned thoroughly with sequential way, as mentioned: (a) Cleaning with 10 g/L of ultrasil P3-11 for 30 min, (b) cleaning with deionized water for 30 min, (c) cleaning with 10 g/L of citric acid for 30 min, and (d) cleaning with deionized water for 1 h. Retentate flow rate of 200 L/h and trans-membrane pressure of 0.8 bar were used during the cleaning with ultrasil and citric acid, whereas trans-membrane pressure of 5 bar and retentate flow rate of 200 L/h were used during the cleaning with water [8]. Membrane hydraulic resistance (*R_m_*) was estimated prior to the experiment, after the experiment, and membrane cleaning. *R_m_* was calculated according to Equation (4). During calculation, dynamic viscosity of deionized water at a temperature of 25 °C was considered.

(4) $$J=\frac{TMP}{\mu \times R_{m}},$$

where *R*_m_ = Membrane hydraulic resistance (m^−1^), μ = Dynamic viscosity of water at temperature 25 °C (Ns/m^2^), *TMP* = Trans-membrane pressure (N/m^2^), and *J* = Water flux (L/(m^2^ h)).

### 2.9. Determination of Protein Concentration 

Concentration of total nitrogen as well as protein in filtrate from muslin cloth, native soybean milk, supernatant after TCA precipitation, and membrane permeate were quantified by the Kjeldahl method [33]. A programmed Kjeldahl nitrogen analyzer, equipped with a block digestion unit (Kjeldatherm^®^; Gerhardt, Königswinter, Germany) was used for this purpose. During conversion of the amount of nitrogen to concentration of protein, a conversion factor of 6.25 was considered. 

### 2.10. Determination of Total Carbohydrate

Concentration of total carbohydrate in filtrate from muslin cloth and native soybean milk were quantified by the phenol sulfuric acid method [41]. Colorimetric determination was performed with a UV-Vis spectrophotometer (EvolutionTM 300; Thermo ScientificTM, Waltham MA, USA). Wavelength 490 nm was used during colorimetric determination. Glucose was used as a reference in the experimental protocol.

### 2.11. Determination of Total Fat 

Concentration of total fat in filtrate from muslin cloth and native soybean milk were quantified by the Gerber method [42]. In a butyrometer, 10 mL of sulfuric acid was mixed with 10.75 mL of filtrate from muslin cloth or native soybean milk. Subsequently, 1 mL of amyl alcohol was added into the butyrometer. Solution was shaken gently and wait until temperature of solution in butyrometer reach to room temperature (~25 °C). Subsequently, the butyrometer was placed in the centrifuge and centrifugation was performed with 1100 rpm for 4 min. Concentration of fat in sample was determined by the position of fat layer in butyrometer.

### 2.12. Determination of Total Antioxidant Capacity 

Antioxidant capacity of native soybean milk, enzyme-hydrolyzed soybean milks, and permeate from membrane were quantified using the ferric reducing ability of plasma method [43]. Colorimetric determination was performed with 593 nm and ascorbic acid as a reference. A UV-Vis spectrophotometer (EvolutionTM 300; Thermo ScientificTM, Waltham MA, USA) was used in the colorimetric determination.

### 2.13. Microbiological Assay 

Antibacterial activity of native soybean milk, enzyme-hydrolyzed soybean milks, and permeate from membrane against Gram-positive *Bacillus cereus* was investigated with agar well method [8]. Microbial strain was collected from the Culture Collection of the Department of Microbiology and Biotechnology, Szent István University, Hungary. Freshly prepared culture of *B. cereus* from liquid broth (8 × 10^6^ colony-forming units/mL) was spread on the soybean casein digestive agar medium with a laboratory glass spreader. Agar wells with the diameter of 5 mm were filled with 100 μL of native soybean milk, enzyme-hydrolyzed soybean milks, and permeate from membrane. Microbial plates were placed at a temperature of 37 °C in a BOD incubator. Zone of inhibition in microbial plate was measured after 48 h of incubation. 

### 2.14. Determination of Molecular Weight of Soybean Milk Proteins and Soybean-Based Peptides by Gel Electrophoresis 

To estimate molecular weight of soybean proteins and soybean-based peptides, the sodium dodecyl sulfate polyacrylamide gel electrophoresis (SDS-PAGE) method was adopted. For this purpose, a vertical electrophoresis system (Bio-Rad mini protein Tetra system, Bio-Rad, Hercules, CA, USA) was used. Concentration of running gel and stacking gel were 15% and 6%, respectively, during the electrophoresis method. The range of molecular weight of standard proteins (precision plus protein standards, Bio Rad, USA) was 250–10 kDa. The samples were prepared by dilution with the sample buffer (Laemmli sample buffer and 2-mercaptoethanole, Bio-Rad, USA). Appropriate diluted 10 µL of soybean milk and enzyme-hydrolyzed soybean milk samples were loaded into the wells. Gel was stained with 0.2% Coomassie Brilliant Blue (R250, Bio-Rad) for 30 min and a gel image was taken using a Gel Doc (Bio-Rad, USA) [44].

### 2.15. Statistical Analysis 

All experiments were performed in triplicate and the mean value with standard deviation (S.D.) was calculated by a Microsoft Excel spread sheet (Microsoft Corporation, Washington WA, USA). Significant differences between different groups were determined by the one-way analysis of variance (ANOVA) method, followed by the Tukey’s post hoc test. The differences were considered significant when *P* < 0.05. The ANOVA test was performed with SPSS 15.0 statistics software (IBM, Armonk NY, USA). 

## 3. Results and Discussion 

The composition of filtrate from muslin cloth was 40 ± 0.96 g/L of protein, 21 ± 0.92 g/L of fat, and 20 ± 0.96 g/L of carbohydrate. After adjustment of pH and concentration of protein, composition of native (unhydrolyzed) soybean milk was 28 ± 0.18 g/L of protein, 15 ± 0.2 g/L of fat, and 14 ± 0.16 g/L of carbohydrate. Native soybean milk was hydrolyzed with different concentrations of papain at different hydrolysis temperature in an advanced-controlled bioreactor, operated with batch-mode. In a later exercise, native soybean milk was used in membrane bioreactor, operated with continuous mode for production of antioxidant and antibacterial peptides.

### 3.1. Enzymatic Hydrolysis of Soybean Milk Proteins with Batch Mode 

**Figure 2:**
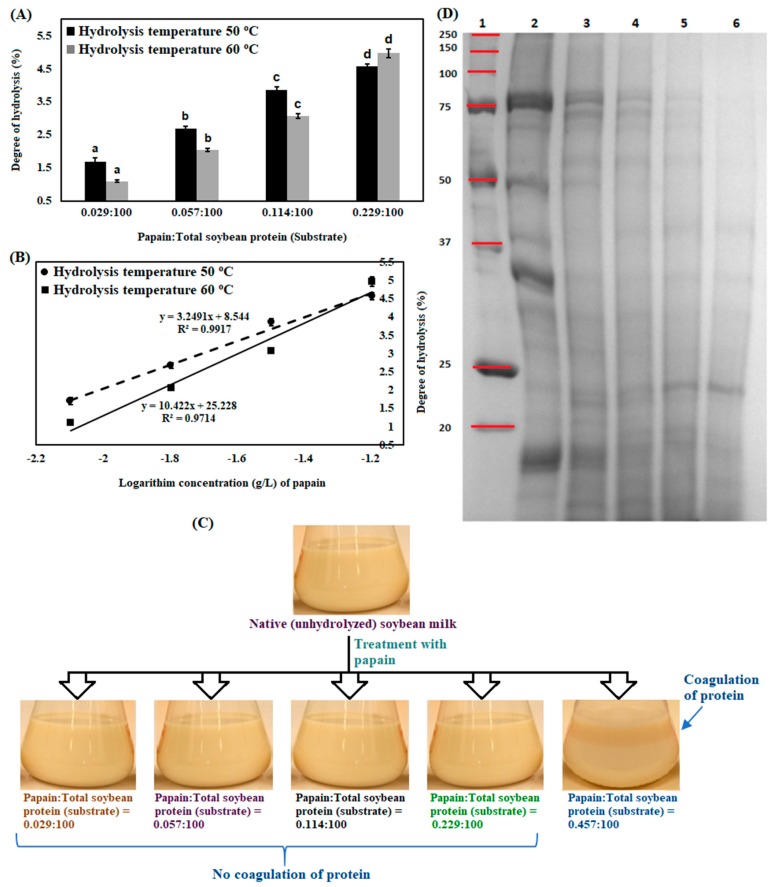
Degree of hydrolysis of soybean milk protein using different concentrations of papain at temperatures of 50 °C and 60 °C (**A**); relation between degree of hydrolysis of soybean milk proteins and concentrations of papain at temperatures of 50 °C and 60 °C (**B**); appearance of soybean milk after enzymatic hydrolysis and deactivation of enzyme (**C**); image of polyacrylamide gel electrophoresis of native soybean milk and enzyme-hydrolyzed soybean milks; lane 1: Marker protein, lane 2: Native (unhydrolyzed) soybean milk, Lane 3: Soybean milk with papain, where papain:total soybean protein (substrate) = 0.029:100, Lane 4: Soybean milk with papain, where papain:total soybean protein (substrate) = 0.057:100, Lane 5: Soybean milk with papain, where papain:total soybean protein (substrate) = 0.114:100; and Lane 6: Soybean milk with papain, where papain:total soybean protein (substrate) = 0.229:100 (**D**).

In Figure 2A, degree of hydrolysis of soybean milk proteins with different concentrations of papain is represented. 

From the figure, it is clear that degree of hydrolysis of soybean milk proteins increased with enzyme concentration (*P* < 0.05). Higher concentration of enzyme triggered more available specific peptide bonds in native protein molecule and promoted to release peptides. Degree of hydrolysis of soybean milk proteins was 1.7% (S.D. ± 0.1) when enzyme and substrate ratio was 0.029:100 and it increased ~2.7-fold due to the increase of enzyme and substrate ratio 0.229:100 at a temperature of 50 °C. Also, the degree of hydrolysis of soybean milk proteins increased at a temperature of 60 °C. Degree of hydrolysis of soybean milk proteins was 1.1% (S.D. ± 0.04) when enzyme and substrate ratio was 0.029:100 and it became 4.9% (S.D. ± 0.13) when enzyme and substrate ratio was 0.229:100 at a temperature of 60 °C. A similar type of observation was also noticed in other hydrolysis temperatures (figure not shown). 

It has been reported that there is no general agreement of the best method/protocol for determining degree of hydrolysis of protein because it depends on cases. The value of degree of hydrolysis of proteinaceous substance depends on several factors, such as the type of enzyme, activity of enzyme, presence of peptide bonds in substrate, hydrolysis time, reaction temperature and pH, and analytical method for the determination of degree of hydrolysis; results are not directly comparable in all situations. Protocols for determining the degree of hydrolysis are categorized in four classes, such as (a) titration of released protons, produced during hydrolysis reaction by pH-STAT method; (b) determination of free α-amino groups in hydrolysis reaction mixture by 2,4,6-trinitro-benzenesulfonic acid (TNBS) or o-phthaldialdehyde (OPA); (c) titration of released protons, produced by amino acids and formaldehyde reaction; and (d) determination of TCA-soluble nitrogen in hydrolysis reaction mixture. However, while pH-stat method was frequently used to determine degree of hydrolysis, its accuracy depends on three major factors: (a) Type of hydrolytic enzyme, (b) size of the hydrolyzed peptides, and (c) reaction temperature [34]. The TNBS, OPA, and formaldehyde titration methods are based on measurement of amino groups, produced during enzymatic hydrolysis of proteins. In TNBS and OPA methods, it is assumed that all derivatized N-terminal amino acids are similar, which may offer an inaccurate result. Furthermore, hazardous chemicals are produced in TNBS method and its prolonged storage is not recommendable [45]. Formaldehyde titration method is not acceptable when histidine, proline, and hydroxyproline are produced in the enzymatic reaction mixture [46]. Determination of degree of enzymatic hydrolysis of protein by measuring TCA-soluble nitrogen in hydrolysis reaction mixture was frequently used because this method can be employed for quantifying nitrogen as well as concentration of protein in all types of nitrogenous substances. Furthermore, direct correlation between TNBS method and TCA-soluble nitrogen method was proven [47]. Realizing all of these aspects, in our investigation, degree of hydrolysis was measured based on TCA-soluble protein fraction. 

In Figure 2B, it is notified that degree of hydrolysis of soybean milk proteins had a linear relationship, when enzyme and substrate ratio was varied between 0.029:100 to 0.229:100. In that concentration range of enzyme, number of peptide bonds of proteins were cleaved by enzyme in a dose-dependent manner and there was no protein precipitation. In soybean milk, the main proteins are β-conglycinin (7S), glycinin (11S), and oleosin (oil body proteins). Protein coagulation was observed when enzyme and substrate ratio was 0.457:100 (Figure 2C). Soybean milk proteins β-conglycinin (7S) and glycinin (11S) are present with globulin association in soybean milk. Soybean milk protein, oleosin, is adsorbed on the surfaces of oil bodies and exists with globular form in soybean milk. Oil bodies in soybean milk are present in a stable manner because they are encapsulated by phospholipid and oleosin. Papain digestion of soybean milk proteins, mainly oil body-stabilizing oleosin, promoted oil body agglomeration with proteins by lipophilic bonding and subsequently created their precipitation [48,49].

Furthermore, hydrolysis of soybean milk proteins was evaluated with SDS-PAGE (Figure 2D). Original image of SDS-PAGE is represented in supplementary section (Figure S1). According to the literature, soybean milk protein β-conglycinin (7S) has α’, α, and β subunits with molecular weights of approximately 81 kDa, 74 kDa, and 50 kDa, respectively [50]. Soybean protein glycinin (11S) has subunits with molecular weight of approximately 14 kDa (basic subunit) and 35 kDa (acidic subunit) [51]. Another soybean protein oleosins (basic nature) have molecular weights of approximately 24 kDa and 18 kDa [52]. In the gel image, subunits are clearly visible because electrophoresis was conducted in the presence of β-mercaptoethanol and sodium dodecyl sulfate. It is observed that large molecular weight protein subunits are converted to low molecular weight peptides with the increase of enzyme concentration. For example, 75 kDa protein subunit is completely hydrolyzed when enzyme and substrate ratio is increased from 0.029:100 to 0.229:100, 50 kDa protein subunit is totally hydrolyzed when enzyme and substrate ratio is increased from 0.029:100 to 0.114:100, and 35 kDa protein subunit is completely hydrolyzed when enzyme and substrate ratio is increased from 0.029:100 to 0.057:100. On the other hand, glycinin subunit with molecular weight 14 kDa is retained (no degradation) even when enzyme and substrate ratio is 0.229:100. This might be due to the fact that 14 kDa subunit (basic group of glycinin) was located at internal position of glycinin complex and had received less chance to participate in enzymatic hydrolysis reaction. Similar observations were reported by other investigators [46]. From the gel image, it is also observed that oleosin with molecular weight 24 kDa is not hydrolyzed when enzyme and substrate ratio is increased from 0.029:100 to 0.229:100. It may be that low concentration of papain and short reaction time are insufficient to hydrolyze oleosins. Oleosin with molecular weight 18 kDa was hydrolyzed when enzyme and substrate ratio is increased from 0.029:100 to 0.229:100. In the gel image, protein bands in lane 6 are lighter compare to lane 3, lane 4, and lane 5 because a large number of proteins and subunits of proteins were hydrolyzed to low molecular weight peptides due to application of higher concentration of papain (enzyme and substrate ratio 0.229:100) in enzymatic hydrolysis of soybean milk protein. Perhaps, low molecular weight peptides were eluted from the gel. 

**Figure 3:**
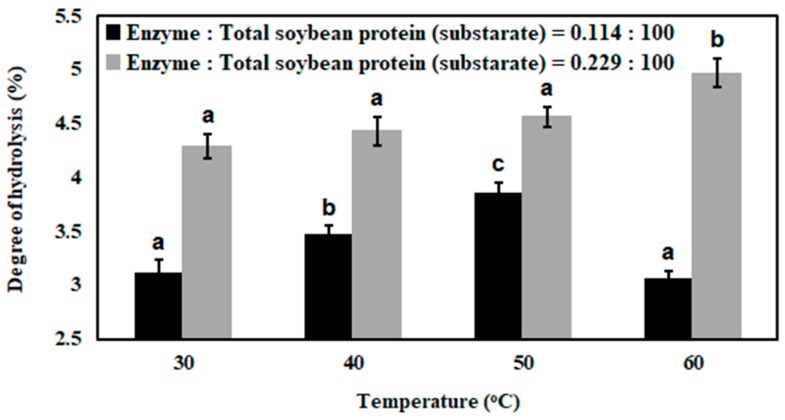
Degree of hydrolysis of soybean milk proteins with different concentrations of papain and hydrolysis temperatures.

In Figure 3, degree of hydrolysis of soybean milk proteins at different reaction temperatures and concentrations of enzyme are represented. 

In the present hydrolysis reaction, degree of hydrolysis of soybean milk proteins was increased with increase of reaction temperature 30 °C to 50 °C (*P* < 0.05), and subsequently it was reduced (*P* < 0.05) at a temperature of 60 °C, when hydrolysis reaction was performed with enzyme and substrate ratio 0.114:100. Furthermore, degree of hydrolysis of soybean milk proteins was increased (*P* < 0.05) with increase of reaction temperature from 30 °C to 60 °C, when hydrolysis reaction was performed with enzyme and substrate ratio 0.229:100. However, this change was ~1.2-fold. In Figure 2, it can also be observed that degree of hydrolysis was lower, when hydrolysis reactions were performed with enzyme and substrate ratios 0.029:100, 0.057:100, and 0.114:100 at a temperature of 60 °C. Therefore, it may be that both temperature and amount of enzyme had a great influence in enzymatic hydrolysis of soybean milk proteins. In an enzymatic reaction, collision frequency between substrate and enzyme as well as activation energy are influenced by reaction temperature. As a consequence, turnover number of enzyme (*k*_cat_) is increased. It influences the increase of reaction rate as well as degree of hydrolysis. Besides activation of enzyme, reaction temperature also influences inactivation of enzyme, which affects the rate of enzymatic reaction as well as degree of hydrolysis [53,54]. At higher temperature, reversible reaction may reduce the activity of enzyme over time [55]. Other possible reasons of inactivation of an enzyme are compaction of the enzyme molecule, which may occurr by the generation of charged molecules around the enzyme and dissociation of enzyme structure at high temperatures [8,56]. 

In the present experiment, soybean milk, containing 28 g/L of total protein, was treated with different concentrations of papain at different temperatures, ranging 30–60 °C for 10 min and, subsequently, inactivation of enzyme was performed with a temperature of 70 °C for 30 min. We did not find any gelation, aggregation, and precipitation of proteins after performing enzymatic hydrolysis and inactivation of enzyme. It was reported that at a temperature of 70 °C and pH 7, β-conglycinin may present in nucleation stage, and nucleation can produce aggregates, but they cannot precipitate (condensate) due to weak interaction between them. Contradictorily, monomers of glycinin produce aggregate and subsequently create insoluble aggregate when soybean milk is treated above the temperature of 75 °C. β-conglycinin in soybean milk at pH 7 reduces aggregation of glycinin and increases the solubility of glycinin. At a temperature of 70 °C, subunits of β-conglycinin turn to unfold and the hydrophobic groups of β-conglycinin might occupy the surface of glycinin, which leads to the termination of the condensation among the aggregates and, subsequently, protein precipitation [57]. Furthermore, it has been reported that heat treatment of soymilk with a temperature of 70 °C to 80 °C might influence denaturation of only β-conglycinin, but not glycinin [58]. As soybean milk proteins such as β-conglycinin and glycinin are sensitive at a temperature of ~75 °C, we used a temperature of 70 °C for inactivation of enzyme and reduced the chance of protein precipitation. While in some research articles it is mentioned that papain has some residual activity at a temperature of 70 °C [37,38,59], in our experiment, we found that after the inactivation step, there was no hydrolysis of proteins over time. The turnover number (*k*_cat_) and decay constant (*k*_d_) of enzymes depend on reaction temperature, amount of enzymes, and time [38,59]. As we used quite a low concentration of enzyme (enzyme and substrate ratio 0.029:100–0.229:100) in the hydrolysis reaction, it may be that after the inactivation step, there was no residual enzyme in reaction environment.

### 3.2. Membrane Bioreactor with Continuous Mode 

**Figure 4:**
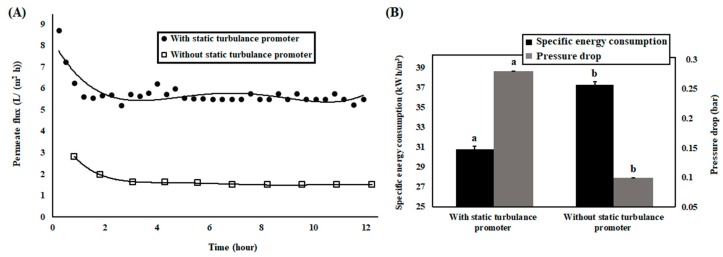
Membrane permeate flux with process time in absence and presence of static turbulence promoter (**A**), specific energy consumption and pressure drop in membrane separation process due to absence and presence of static turbulence promoter (**B**).

In Figure 4A, permeate flux during whole operational process is presented to describe the effect of static turbulence promoter on filtration. 

It is observed that, initially, *permeate flux* was high and was subsequently reduced and became asymptotic with filtration time. As the concentrations of unhydrolyzed proteins and peptides (molecular weight higher than 5 kDa) in the bioreactor were high, the concentration polarization, created by enzyme, unhydrolyzed proteins, or peptides on membrane surface took place during filtration. As the membrane separation process was performed with continuous mode, constant steady flux was achieved after flux declination. It was found that application of static turbulence promoter had a positive influence on permeation. When the static turbulence promoter was not used inside of the membrane tube, initial permeate fluxes were 2.8 L/(m^2^ h) and this value changed to 8.7 L/(m^2^ h) when the static turbulence promoter was applied inside of the membrane tube. At a constant trans-membrane pressure of 3 bar and a retentate flow rate of 100 L/h, the static turbulence promoter created more pressure drop at two opposite ends of membrane module, whereas the pressure drop was negligible when the filtration process was performed without that device (Figure 4B). The static turbulence promoter created turbulence in working fluid, offered tangential and centrifugal forces, increased velocity, and vorticity of fluid across the membrane surface, which reduced deposition of solutes on membrane surface, membrane gel layer resistance, and increased permeate flux. As the static turbulence promoter offered higher permeate flux, specific energy consumption during the static turbulence promoter-implemented filtration process was lower compared to the filtration without that device (Figure 4B). 

After membrane compaction, *membrane hydraulic resistance with deionized water was* determined to be 1.432 × 10^13^ m^−1^. After the experiment with static turbulence promoter and without static turbulence promoter, this value became 1.432 × 10^14^ m^−1^ and 9.922 × 10^15^ m^−1^, respectively. During the membrane filtration process, concentration polarization took place on membrane surface, which increased membrane gel-layer resistance. As a result, membrane hydraulic resistance was increased. Several investigators reported that static turbulence promoter has a positive effect on membrane cleaning [60,61]. Therefore, after experiment membrane was always cleaned with the mentioned protocol in the presence of static turbulence promoter. In every case, the value of permeate flux of deionized water returned to its original value after cleaning of membrane. As the static turbulence promoter created high tangential force along with driving force, offered by trans-membrane pressure on fluid, deposited solutes were removed from membrane surface and membrane pores. 

Realizing the great potentiality of static turbulence promoter in filtration process, antioxidant capacity and antibacterial activity of peptides, obtained by static turbulence promoter-implemented filtration process, have been investigated.

### 3.3. Antioxidant Capacity

**Figure 5:**
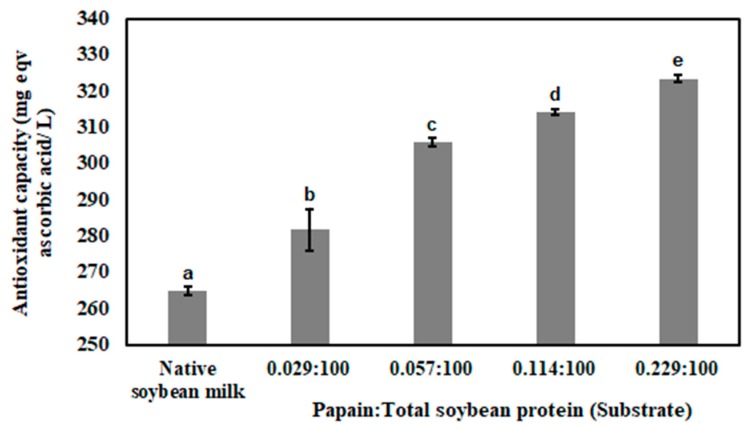
Antioxidant capacity of native soybean milk and papain-treated soybean milks, prepared with different concentrations of papain at temperature 50 °C.

Antioxidant capacities of soybean milk and enzyme-treated soybean milks are represented in Figure 5.

Antioxidant capacity of native soybean milk was 264.8 ± 1.16 mg eqv. ascorbic acid/L and it was increased (*P* < 0.05) in enzyme-treated soybean milks. Papain prefers to cleave at (hydrophobic amino acid)-(Arg or Lys or Glu or His or Gly or Tyr) in protein structure. Antioxidant capacity of papain-treated soybean milk was increased in a dose-dependent manner. This can be justified by the fact that with the increase of the concentration of papain, more amounts of peptide bonds in soybean milk proteins were hydrolyzed and hydrophobic amino acids were exposed. The hydrophobic amino acids offer better reducing activity towards ferric ions and suppress their pro-oxidant activity than native proteins [24,25,62].

**Figure 6:**
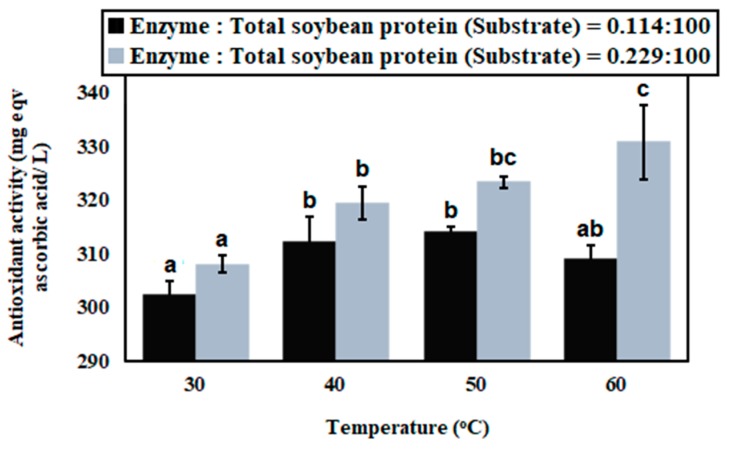
Antioxidant capacity of papain-treated soybean milks, prepared with different concentrations of papain and hydrolysis temperatures.

In Figure 6, antioxidant capacities of enzyme-treated soybean milks, prepared at different hydrolysis temperatures, are represented. 

It was found that antioxidant capacity was increased due to increase of temperature from 30 °C to 50 °C (*P* < 0.05) and, subsequently, it was reduced at a temperature of 60 °C when the hydrolysis reaction was performed with enzyme and substrate ratio 0.114:100; whereas antioxidant capacity was increased with increase of temperature, i.e., 30 °C to 60 °C (*P* < 0.05) when hydrolysis reaction was performed with enzyme and substrate ratio 0.229:100. A similar type of trend was observed in case of estimation of degree of hydrolysis, represented in Figure 3. Hydrophobic amino acids in terminal end of peptides were produced by enzymatic hydrolysis of soybean proteins. Hydrophobic amino acids in peptide chain offer antioxidant capacity. The degree of hydrolysis of soybean milk proteins was strongly influenced by the concentration of enzyme and hydrolysis temperature. Comprehensive justification is mentioned in Section 3.1. Therefore, it may be that degree of hydrolysis and antioxidant capacity of peptides are directly corelated. 

**Figure 7:**
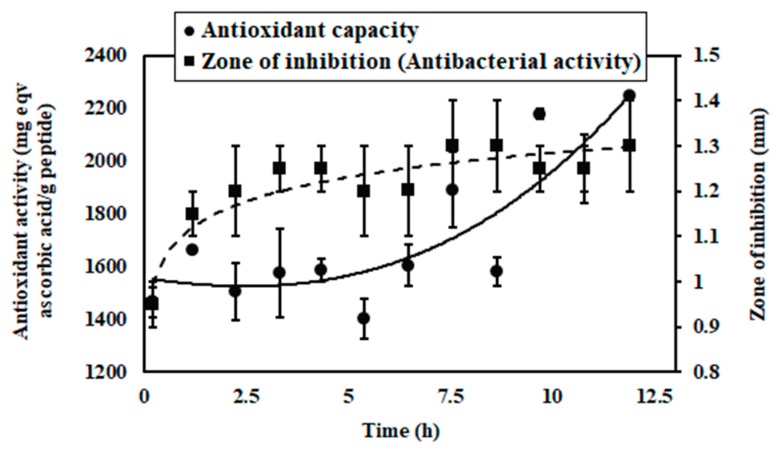
Antioxidant capacity and zone of inhibition of membrane permeate in different process time.

In Figure 7, antioxidant capacity of peptides in membrane permeate, collected in a fractional way during the whole process (12 h), is represented. 

It was found that antioxidant capacity in membrane permeate, collected at different fractions, were increased with time progress. This can be justified by the fact that over time, more soybean milk proteins were hydrolyzed and permeated through membrane pores in a continuous way. Continuous feeding of soybean milk to bioreactor and high concentration of enzyme, i.e., 0.9 g/L promoted the production of a higher quantity of peptides with molecular weight lower than 5 kDa and antioxidant capacity. 

### 3.4. Antibacterial Activity

In batch-mode hydrolysis reaction, it was found that antibacterial activity *in* enzyme-treated soybean milk *was significantly increased* (*P* < 0.05) compare to native soybean milk. The values of zone of inhibition were 2 ± 0.02 mm and 8 ± 0.07 mm when enzyme and substrate ratios were 0.029:100 and 0.229:100, respectively. The antibacterial activity of enzyme-treated milks may be explained by several biochemical phenomena. When soybean milk was treated with papain, soybean milk proteins are converted to peptides with acidic or alkaline amino acids in C or N terminal end. Hydrophilic, hydrophobic, or amphiphatic peptides interact with bacterial peptidoglycan, which might facilitate penetration of peptides to a biotic phase. In peptidoglycan, anionic teichoic acid may trap antibacterial peptides or act as a ladder to facilitate the interaction between peptides and bacterial cytoplasmic membrane. Interaction between antibacterial peptide with cytoplasmic membrane frequently leads to lipid segregation in cell membrane, which increases membrane permeability, inhibits cell division, or leads to delocalization of essential membrane proteins. Furthermore, it has been reported that antibacterial peptides form a complex with the precursor molecules of cell wall components, which might promote pore formation and, subsequently, membrane disruption. Stable pores in the cell membrane facilitate permeabilization of cellular content into the outer environment as well as cellular death [63,64]. In this context, Patrzykat and Douglas reported that antibacterial activity of peptides not only depends on their binding ability with bacterial cell membrane. Amino acid sequence in peptide, concentration of peptide, and chemical composition of bacterial cell membrane influence the antibacterial activity of peptides [65]. 

Antibacterial activity (zone of inhibition) from permeate of membrane against *Bacillus cereus was also noted (Figure 7).* It was found that the zone of inhibition by permeate from membrane, collected at different fractions during the whole period, increased over time. As the membrane bioreactor was operated with a continuous supply of soybean milk into the bioreactor and sufficient amount of papain, more soybean milk proteins were hydrolyzed over time and permeated through porous channel of membrane in a continuous way. Every fraction of permeate from membrane offered a low zone of inhibition compare to batch-mode hydrolysis reaction mixture because a low quantity of antimicrobial peptides was permeated through membrane pores in every time intervals. Cumulative number of peptides, produced in batch-mode enzymatic hydrolysis reaction, offered a comparatively high zone of inhibition. In another investigation, we found that peptides with molecular weight lower than 5 kDa, produced by tryptic hydrolysis of defatted soybean meal protein, had antibacterial activity against Gram-positive *B. cereus* [8]. However, sources of peptides were different, i.e., soybean milk and defatted soybean meal, and they were prepared by enzymatic hydrolysis with papain and trypsin, respectively; their antimicrobial activity on test microorganism were similar. This result may encourage future researchers to identify the amino acid sequence in peptide chain and subsequently, may boost the development of natural antibacterial biomolecules.

## 4. Conclusions

Preparation of bioactive peptides from common food stuffs through an environmentally benign biochemical route is considered a promising approach in biopharmaceutical and food industries. In the present investigation, antioxidant and antibacterial peptides were produced from soybean milk by a membrane bioreactor (a bioreactor with an external crossflow membrane separation unit). To understand the protein hydrolysis process, soybean milk proteins were initially hydrolyzed by papain in an advanced-controlled bioreactor, operated with batch mode. The degree of hydrolysis of soybean milk proteins and antioxidant capacity were increased (*P* < 0.05) with increase of enzyme concentration. The degree of hydrolysis of soybean milk proteins showed an increasing trend (*P* < 0.05) when reaction temperature was increased from 30 °C to 60 °C, and enzyme and substrate ratio was 0.229:100. Contradictorily, the degree of hydrolysis of soybean milk proteins was reduced when hydrolysis reaction was performed with enzyme and substrate ratio 0.114:100, and reaction temperature was 60 °C. This might be due to inactivation of enzyme. Antibacterial activity of enzyme-hydrolyzed soybean milk against *B. cereus* was increased compared with native soybean milk. Furthermore, antioxidant and antibacterial peptides were produced from soybean milk by a membrane bioreactor, operated for 12 h with continuous feeding. It was found that permeate flux was increased when the static turbulence promoter was applied at constant operational trans-membrane pressure of 3 bar and a retentate flow rate of 100 L/h in the membrane separation process. Furthermore, energy consumption was reduced due to the application of static turbulence promoter in filtration process.

Preparation of antioxidant- and antibacterial- peptides from soybean milk through the enzymatic and membrane routes may be considered as a first attempt, to the best of our knowledge and literature survey. It is expected that the result will be useful to scale-up the process and mitigate the limitations of the production of bioactive peptides from soybean milk or other protein sources on an industrial scale. Therefore, it may be that this investigation is of great importance to future research communities and biopharmaceutical and food industries.

## Figures and Tables

**Table 1:** Specification of membrane and static turbulence promoter.

Characteristics of Tubular Membrane		Characteristics of Twisted Tape Static Turbulence Promoter	
Pore size	20 nm	Aspect ratio	2
Length	250 mm	Diameter	6.5 mm
Inner diameter	7 mm	Total length	241 mm
Outer diameter	10 mm	Pitch length	13.2 mm
Active surface area	5 × 10^−3^ m^2^	Number of mixing elements	36
Active layer	Titanium oxide	Thickness	1.2 mm
Support layer	Aluminum oxide	Material	Stainless steel (SS316)

## Supplementary Materials

The following are available online at https://www.mdpi.com/2306-5354/7/1/5/s1, Figure S1: Image of polyacrylamide gel electrophoresis of native soybean milk and enzyme-hydrolyzed soybean milks.
