# Supplementary material for: Antioxidant and Antibacterial Peptides from Soybean Milk through Enzymatic- and Membrane-Based Technologies
Source: Bioengineering (Basel). 2019 Dec 28;7(1):5. doi: 10.3390/bioengineering7010005 (PMC7175099; doi:10.3390/bioengineering7010005)
Supplement: Supplementary file 1 [file bioengineering-07-00005-s001.pdf]

bioengineering

MDPI

Article

Antioxidant and Antibacterial Peptides from Soybean Milk through Enzymatic- and Membrane-Based Technologies

Arijit Nath [1,2], Geremew Geidare Kailo [1], Zsuzsanna Mednyánszky [3], Gabriella Kiskó [4], Barbara Csehi [5], Klára Pásztorné-Huszár [5], Renáta Gerencsér-Berta [2], Ildikó Galambos [2], Emília Pozsgai [2], Szilvia Bánvölgyi [1] and Gyula Vatai [1,*]

[1] Department of Food Engineering, Faculty of Food Science, Szent István University, Ménesi st 44, Budapest, HU-1118, Hungary; arijit0410@etk.szie.hu (A.N.); geidaregeremew@gmail.com (G.G.K.); szbanvolgyi.uni.szie@gmail.com (S.B.);

[2] Soós Ernő Water Technology Research and Development Center, University of Pannonia, Zrínyi M. u. 18, H-8800 Nagykanizsa, Hungary; berta.renata@sooswrc.hu (R. G-B.); galambos.ildiko@sooswrc.hu (I.G.); pozsgai.emilia@sooswrc.hu (E.P.);

[3] Department of Food Chemistry and Nutrition, Faculty of Food Science, Szent István University, Budapest, Somlói st 14-16, Budapest, HU-1118, Hungary; Mednyanszky.Zsuzsanna@etk.szie.hu;

[4] Department of Food Microbiology and Biotechnology, Faculty of Food Science, Szent István University, Budapest, Somlói st 14-16, Budapest, HU-1118, Hungary; Kisko.Gabriella@etk.szie.hu;

[5] Department of Refrigeration and Livestock Product Technology, Faculty of Food Science, Szent István University, Ménesi st 43-45, Budapest, HU-1118, Hungary; Csehi.Barbara@etk.szie.hu (B.C.); Pasztorne.Huszar.Klara@etk.szie.hu (K. P-H.);

* Correspondence: Vatai.Gyula@etk.szie.hu; Tel.: +3613057112; Fax: +36-1-305-6323

Supplementary Materials

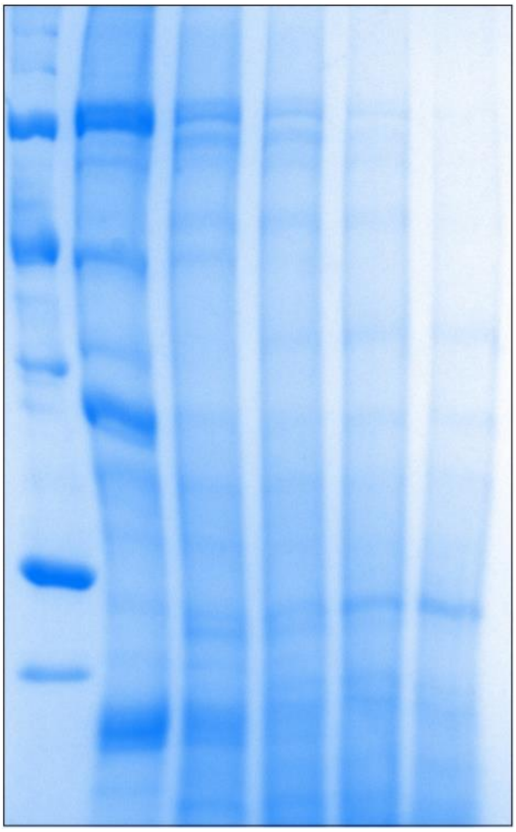

Figure S1. Image of polyacrylamide gel electrophoresis of native soybean milk and enzyme-hydrolyzed soybean milks.
